# Replication of the association of HLA-B7 with Alzheimer's disease: a role for homozygosity?

**DOI:** 10.1186/1742-2094-3-33

**Published:** 2006-12-18

**Authors:** Donald J Lehmann, Martin CNM Barnardo, Susan Fuggle, Isabel Quiroga, Andrew Sutherland, Donald R Warden, Lin Barnetson, Roger Horton, Stephan Beck, A David Smith

**Affiliations:** 1 The Oxford Project to Investigate Memory and Ageing (OPTIMA), University Department of Pharmacology & Radcliffe Infirmary, Oxford, UK; 2 Oxford Centre for Gene Function, University Department of Physiology, Anatomy & Genetics, Parks Rd, Oxford OX1 3PT, UK; 3 Nuffield Department of Surgery, University of Oxford, John Radcliffe Hospital, Oxford OX3 9DU, UK; 4 Oxford Transplant Centre, Churchill Hospital, Oxford OX3 7LJ, UK; 5 Immunogenomics Laboratory, Wellcome Trust Sanger Institute, Genome Campus, Hinxton, Cambridge CB10 1SA, UK

## Abstract

**Background:**

There are reasons to expect an association with Alzheimer's disease (AD) within the *HLA *region. The *HLA-B *&* C *genes have, however, been relatively understudied. A geographically specific association with HLA-B7 & HLA-Cw*0702 had been suggested by our previous, small study.

**Methods:**

We studied the *HLA-B *&* C *alleles in 196 cases of 'definite' or 'probable' AD and 199 elderly controls of the OPTIMA cohort, the largest full study of these alleles in AD to date.

**Results:**

We replicated the association of HLA-B7 with AD (overall, adjusted odds ratio = 2.3, 95% confidence interval = 1.4–3.7, *p *= 0.001), but not the previously suggested interaction with the ε4 allele of apolipoprotein E. Results for HLA-Cw*0702, which is in tight linkage disequilibrium with HLA-B7, were consistent with those for the latter. Homozygotes of both alleles appeared to be at particularly high risk of AD.

**Conclusion:**

HLA-B7 and HLA-Cw*0702 are associated with AD in the Oxford population. Because of the contradictions between cohorts in our previous study, we suggest that these results may be geographically specific. This might be because of differences between populations in the structure of linkage disequilibrium or in interactions with environmental, genetic or epigenetic factors. A much larger study will be needed to clarify the role of homozygosity of *HLA *alleles in AD risk.

## Background

There are grounds to suspect a connection between Alzheimer's disease (AD) and variation in the major histocompatibility complex at the chromosomal region, 6p21.3. AD is characterised by chronic inflammation and altered immune function, including activation of immunocompetent glia expressing high levels of human leukocyte antigen (HLA) molecules, complement and pro-inflammatory cytokines [1]. Many of these proteins are encoded in the region. Genome scans [2,3] have implicated the region. Long-term use of non-steroidal anti-inflammatory drugs is associated with reduced risk of AD [4-6].

The region has proved a challenge for the study of disease associations, because it is highly variable, with a complex structure of linkage disequilibrium. However, it is also true that, apart from the study of certain genes, e.g. *TNF *[7], and alleles, e.g. HLA-A2 [8], most studies of *HLA *genes in AD have been seriously underpowered. This is particularly so for *HLA-B *and *C *(see Discussion). Our own previous study [9], with 55 cases of AD and 73 controls from the Oxford Project to Investigate Memory and Ageing (OPTIMA), suggested an association with AD of two alleles in linkage disequilibrium with each other, HLA-B7 and HLA-Cw*0702, especially in people without the ε4 allele of apolipoprotein E (*APOE*4). As that association was not replicated in two other cohorts involved in the study [9], it remains possible that these contrasts were due to geographical differences, for instance in the fine structure of linkage disequilibrium or in interactions with other risk factors (see Discussion).

We now examined *HLA-B *and *C *alleles in a further 141 cases of AD and 143 controls from the longitudinal, observational cohort of OPTIMA. Thus, altogether 196 cases of AD and 199 controls were studied, i.e. including 55 cases and 56 controls from our previous study [9] (17 of the 73 controls from that study now have other diagnoses, e.g. mild cognitive impairment, and have therefore been excluded from analysis). We aimed to replicate the association with HLA-B7 and HLA-Cw*0702 and to examine other alleles at those loci.

## Methods

All 196 cases of AD (110 women) and 199 controls (107 women) were Caucasians in OPTIMA, drawn from the Oxford region and followed with detailed annual assessments [10] for up to 15 years. The cohorts for both our studies were drawn from the same Oxford population and ascertained in a similar way. OPTIMA protocols [10] have been approved by the Central Oxford Ethics Committee No 1656. Mean onset age of AD was 70.5 (± 8.9) years and of death or last examination of controls was 76.7 (± 9.2) years. Of the AD cases, 122 were neuropathologically confirmed by CERAD criteria [11] (104 "definite" and 18 "probable") and 74 were diagnosed "probable AD" by NINCDS-ADRDA criteria [12]. Possible autosomal dominant cases were excluded, based on family history. All 199 controls were without cognitive impairment and with CAMCOG scores [13] > 80.

*HLA-B *and *Cw *genotyping was performed by PCR-SSP using a modification of the Phototyping method [14]. Standard PCR methods were used for *APOE*4 [15]. All genotyping was undertaken blind to diagnosis. Unadjusted *p *values were by Fisher's exact test; odds ratios were also adjusted for age, gender and *APOE*4 status by logistic regression analysis. Potential interactions were examined by logistic regression analysis. Of the 26 studied alleles, 14 had a minor allele frequency > 5%. In the overall analyses of each allele, therefore, a Bonferroni correction factor of 14 was applied, except in the replication study of HLA-B7 and HLA-Cw*0702. In subgroup analyses, stratified by gender and by *APOE*4 status, a correction factor of 14 × 4 = 56 was used.

## Results

### Hardy-Weinberg equilibrium

Out of 52 Hardy-Weinberg analyses (26 analyses of controls and 26 of cases), three resulted in disequilibrium at *p *< 0.05, as expected by chance: *HLA*-B39 controls, *HLA*-B51 cases and *HLA*-B65 cases, each due to a single homozygote of a relatively rare allele. All other alleles were in Hardy-Weinberg equilibrium in both cases and controls (Tables 1 &2).

**Table 1:** *HLA-B *alleles in controls and in Alzheimer's disease

Allele	Homozygotes/heterozygotes/negatives (n)		Allelic frequency (%)		Unadjusted allelic odds ratio of AD (95% CI, *p*^†^)	Hardy-Weinberg equilibrium (*p*^†^)	
	
	Controls	AD	Controls	AD		Controls	AD
HLA-B7	0/42/157	7/58/131	10.6	18.4	1.9 (1.3–2.9, 0.002)	0.1	0.85
HLA-B8	5/49/145	6/45/145	14.8	14.5	1.0 (0.7–1.45, 0.9)	0.7	0.3
HLA-B18	1/16/182	1/11/184	4.5	3.3	0.7 (0.35–1.5, 0.5)	0.3	0.08
HLA-B27	0/11/188	0/18/178	2.8	4.6	1.7 (0.8–3.6, 0.2)	0.7	0.5
HLA-B35	0/22/177	1/25/170	5.5	6.9	1.3 (0.7–2.3, 0.5)	0.4	0.9
HLA-B39	1/9/189	0/7/189	2.8	1.8	0.6 (0.25–1.7, 0.5)	0.025	0.8
HLA-B44	3/56/140	7/48/141	15.6	15.8	1.0 (0.7–1.5, 1.0)	0.3	0.3
HLA-B51	0/20/179	1/8/187	5.0	2.6	0.5 (0.2–1.1, 0.09)	0.5	0.01
HLA-B57	0/19/180	1/19/176	4.8	5.4	1.1 (0.6–2.1, 0.75)	0.5	0.5
HLA-B60	1/31/167	0/19/177	8.3	4.8	0.6 (0.3–1.0, 0.06)	0.7	0.5
HLA-B62	1/31/167	0/23/173	8.3	5.9	0.7 (0.4–1.2, 0.2)	0.7	0.4
HLA-B65	0/13/186	1/4/191	3.3	1.5	0.5 (0.2–1.2, 0.2)	0.6	0.0001

AD = Alzheimer's disease, CI = confidence interval ^†^uncorrected

**Table 2:** *HLA-C *alleles in controls and in Alzheimer's disease

Allele	Homozygotes/heterozygotes/negatives (n)		Allelic frequency (%)		Unadjusted allelic odds ratio of AD (95% CI, *p*^†^)	Hardy-Weinberg equilibrium (*p*^†^)	
	
	Controls	AD	Controls	AD		Controls	AD
HLA-Cw1	0/9/190	1/14/180	2.3	4.1	1.85 (0.8–4.2, 0.2)	0.7	0.2
HLA-Cw2	0/15/184	0/16/179	3.8	4.1	1.1 (0.5–2.2, 0.9)	0.6	0.55
HLA-Cw4	1/36/162	1/34/160	9.5	9.2	1.0 (0.6–1.6, 0.9)	0.5	0.6
HLA-Cw5	1/36/162	1/40/154	9.5	10.8	1.1 (0.7–1.8, 0.6)	0.5	0.35
HLA-Cw6	4/31/164	2/31/162	9.8	9.0	0.9 (0.6–1.5, 0.7)	0.09	0.7
*HLA*-Cw*0701	6/62/131	7/56/132	18.6	17.9	1.0 (0.7–1.4, 0.85)	0.7	0.7
*HLA*-Cw*0702	1/46/152	9/58/128	12.1	19.5	1.8 (1.2–2.6, 0.0045)	0.2	0.5
*HLA*-Cw*0704	0/8/191	0/3/192	2.0	0.8	0.4 (0.1–1.4, 0.2)	0.8	0.9
HLA-Cw8	0/17/182	1/11/183	4.3	3.3	0.8 (0.4–1.6, 0.6)	0.5	0.08
HLA-Cw9	0/29/170	0/25/170	7.3	6.4	0.9 (0.5–1.5, 0.7)	0.3	0.3
HLA-Cw10	1/37/161	2/24/169	9.8	7.2	0.7 (0.4–1.2, 0.2)	0.5	0.3
HLA-Cw12	1/11/187	0/8/187	3.3	2.1	0.6 (0.25–1.5, 0.4)	0.08	0.8
HLA-Cw15	0/13/186	0/2/193	3.3	0.5	0.15 (0.03–0.7, 0.007)	0.6	0.9
HLA-Cw16	0/13/186	1/13/181	3.3	3.8	1.2 (0.6–2.5, 0.7)	0.6	0.2

AD = Alzheimer's disease, CI = confidence interval ^†^uncorrected

### Linkage disequilibrium

Four well-known examples of linkage disequilibria were confirmed: HLA-B7 and HLA-Cw*0702 (overall D' = 96%, r^2 ^= 0.82), HLA-B8 and HLA-Cw*0701 (99%, 0.75), HLA-B35 and Cw4 (96%, 0.58), and HLA-B44 and Cw5 (76%, 0.36). Similar patterns were seen both in controls and in cases.

### Possible associations of AD with HLA-B & C alleles

Tables 1 and 2 show the overall results for the 26 studied alleles. Apart from the associations with HLA-B7 and *HLA*-Cw*0702 (see below), there was one other apparently significant association, i.e. with HLA-Cw15, before correction for multiple testing. Subgroup analysis, stratifying by gender and by *APOE*4 status, revealed various other associations before correction: HLA-B27 in *APOE*4 negatives (odds ratio = 2.95, 95% confidence interval = 1.1–7.9, *p *= 0.035); HLA-Cw1 in *APOE*4 negatives (3.4, 1.2–9.6, 0.03) and in men (11.3, 1.4–89, 0.004); HLA-Cw15 in *APOE*4 positives (0.11, 0.01–0.99, 0.03) and in men (0.11, 0.01–0.9, 0.02). There was also a significant interaction between HLA-Cw1 and sex (*p *= 0.03, logistic regression). However, none of these apparently significant results survived Bonferroni correction. Only a weak tendency towards an association with HLA-Cw15 overall remained after correction (*p *= 0.1). All further results reported below relate to HLA-B7 and *HLA*-Cw*0702.

### Replication study of HLA-B7 and HLA-Cw*0702

Table 3 shows the results for HLA-B7 and *HLA*-Cw*0702 by study, i.e. our previous study (included in our 2001 report [9]) and the replication study. The association of HLA-B7 with AD was replicated and that of *HLA*-Cw*0702 tended also to be replicated. As expected, similar overall results were found for the HLA-B7/HLA-Cw*0702 haplotype (data not shown). In view of the consistency between the results of the two studies, we pooled the two datasets for further analysis of HLA-B7 and *HLA*-Cw*0702.

**Table 3:** Associations of AD with HLA-B7 and HLA-Cw*0702 by study

Allele	Study	Proportions of alleles		Adjusted^‡ ^odds ratios of AD (95% CI, *p*)
			
		Controls	AD	
HLA-B7	Previous^†^	10/112	26/110	3.1 (1.2–8.0, 0.02)
	Replication	32/286	46/282	1.9 (1.03–3.4, 0.04)
	All	42/398	72/392	2.3 (1.4–3.7, 0.001)

*HLA*-Cw*0702	Previous^†^	14/112	28/108	2.7 (1.1–6.3, 0.03)
	Replication	34/286	48/282	1.7 (0.95–2.9, 0.08)
	All	48/398	76/390	2.0 (1.3–3.1, 0.003)

^†^results included in our 2001 report [9]

AD = Alzheimer's disease, CI = confidence interval

^‡^for age, gender and the ε4 allele of apolipoprotein E

### Possible interactions of HLA-B7 and HLA-Cw*0702 with other factors

Table 4 shows the associations of AD with HLA-B7 and HLA-Cw*0702 by *APOE*4 status. Although the odds ratios were higher in *APOE*4 negatives than in positives and were only significant in the former, the differences were not significant. Moreover, neither interaction of HLA-B7 nor of *HLA*-Cw*0702 with *APOE*4 was significant (*p *= 0.27 and 0.55, respectively) by logistic regression analysis. Nor were there any significant interactions with age or gender (data not shown).

**Table 4:** Associations of AD with HLA-B7 and HLA-Cw*0702 by *APOE*4 status

Allele	*APOE*4 status	Proportions of alleles		Adjusted^† ^odds ratios of AD (95% CI, *p*)
			
		Controls	AD	
HLA-B7	Positive	14/110	42/246	1.7 (0.8–3.55, 0.19)
	Negative	28/288	30/146	2.8 (1.5–5.2, 0.002)
	All	42/398	72/392	2.3 (1.4–3.7, 0.001)

*HLA*-Cw*0702	Positive	14/110	45/244	1.7 (0.8–3.6, 0.15)
	Negative	34/288	31/146	2.2 (1.2–4.0, 0.01)
	All	48/398	76/390	2.0 (1.3–3.1, 0.003)

AD = Alzheimer's disease, *APOE*4 = the ε4 allele of apolipoprotein E, CI = confidence interval ^†^for age, gender and *APOE*4

### Effects of homozygosity of HLA-B7 and HLA-Cw*0702

Table 5 shows that the odds ratios of AD were much higher for homozygotes than for heterozygotes. In the case of *HLA*-Cw*0702, the odds ratio was only significant for homozygotes.

**Table 5:** Associations of AD with HLA-B7 and HLA-Cw*0702 by zygosity

Allele	Homozygotes/heterozygotes/negatives(n)		Unadjusted^† ^odds ratios of AD (95% CI, *p*) (versus negatives)
		
	Controls	AD	
HLA-B7	0/42/157	7/58/131	Heterozygotes: 1.7 (1.02–2.7, 0.04)
			Homozygotes: 18.0 (1.6–202, 0.0045)
*HLA*-Cw*0702	1/46/152	9/58/128	Heterozygotes: 1.5 (0.9–2.4, 0.09)
			Homozygotes: 10.7 (1.6–72.0, 0.007)

AD = Alzheimer's disease, CI = confidence interval.

^†^Analyses were unadjusted since there were too few homozygotes for regression analysis

### The effect on onset age

Neither HLA-B7 nor *HLA*-Cw*0702 was associated with onset age of AD (data not shown).

## Discussion

We suggest that the only results meriting further scrutiny are those for HLA-B7 and HLA-Cw*0702 and possibly the potentially reduced risk associated with HLA-Cw15. All other apparently significant results, i.e. before correction, are probably due to multiple testing. However, our replication of the HLA-B7 finding, which was significant in both studies, implies that that allele is indeed associated with increased risk of AD in the Oxford population. The results for *HLA*-Cw*0702 were consistent with those for HLA-B7. Because of the tight linkage disequilibrium between these two alleles and also their similar frequency, we cannot be certain which is closer to the true risk locus.

### Previous studies

To our knowledge, there have been 17 previous AD association studies that included *HLA-B *or *C *alleles or both. Fifteen of those were before 1990, based on phenotyping methods, using AD cases that were nearly all clinically diagnosed, usually by an unspecified method. Two of those early studies [16,17] reported an increased risk of AD associated with HLA-B7. Of the 17 studies, only three had more than 60 AD cases: one Japanese study [18] (122 AD cases) and two Caucasian, Middleton et al 1999 [19] (95 AD cases) and our previous study [9] (299 AD cases from three cohorts; however, full *HLA*-*B *&*C *typing was only performed in the OPTIMA cohort, with 55 AD cases). Thus surprisingly, the present study is the largest, full study of *HLA*-*B *&*C *genes so far, and the only one large enough to examine the effects of homozygosity (Table 5).

### Population-specific risk

Since the association with HLA-B7 was not replicated in the two other cohorts in our previous study [9], one mainly from Cambridge and the other from Montreal, the association reported here is likely to be geographically specific, although chance variation doubtless also played a part. This geographical specificity could be due to differences in the fine structure of linkage disequilibrium between populations or to different interactions with other risk factors: environmental, genetic or epigenetic. Epigenetic patterns, such as DNA methylation and chromatin modifications, affect gene expression and are thought to be stably maintained during somatic cell divisions, i.e. they are mitotically heritable. But they vary between tissues and between populations and degenerate with age [20,21]. Most complex diseases are age-related. Thus epigenetic patterns, as well as genetic and environmental factors, will contribute to variation between populations.

### Potential interactions

In the present study, we found no interactions of HLA-B7 or of *HLA*-Cw*0702 with age, gender or *APOE*4; we consider the apparent difference between *APOE*4 positives and negatives (Table 4) to be most likely due to chance. This result thus contradicts our previous suggestion [9] of an interaction with *APOE*4. Large numbers, however, are needed to demonstrate interactions.

### Homozygosity

The odds ratios for HLA-B7 and *HLA*-Cw*0702 homozygotes appear striking (Table 5). However, they are partly due to partial (i.e. not significant at 0.05) Hardy-Weinberg disequilibrium in controls (*p *= 0.1 for HLA-B7, *p *= 0.2 for HLA-Cw*0702). Nevertheless, if one were artificially to restore precise Hardy-Weinberg equilibrium to controls, the odds ratios for homozygotes of each allele would still be approximately 4 and those for heterozygotes would remain just below 2. This would still suggest a codominant or dose effect of these alleles. Incidentally, one study [22] reported an association of homozygotes, not of heterozygotes of HLA-A2 with earlier onset of AD; however, HLA-A2 is on a different haplotype from HLA-B7/*HLA*-Cw*0702/HLA-A3.

Alternatively, could the lack of homozygotes in controls (Table 5) be a true effect due to their selective vulnerability, not only to AD? Low natural killer (NK) cell activity has been associated with homozygosity for both the HLA-B7/*HLA*-Cw*0702 and the HLA-B8/*HLA*-Cw*0701 haplotypes in Caucasians [23,24] and for HLA-B7 in Chinese [25]. This low NK cell activity may be due to a recessive gene or variable site in linkage disequilibrium with these haplotypes. In our previous study [9], AD was associated with HLA-B7 in one cohort and with HLA-B8 in another, mainly or only in subjects without *APOE*4.

Homozygosity at *HLA *class I loci has been associated with greater susceptibility to viral infection [26,27], perhaps partly due to an inadequate defence by NK cells [24]. However, this effect was not seen in our cohort, since there was no overall shortage of homozygotes, only in controls. Alternatively therefore, could it be that low NK cell activity increases vulnerability to AD?

### NK cells and AD

NK activity has been rather little studied in AD. However, there may be changes in the peripheral activity of NK cells in AD, although reports conflict [28-31]. It has been proposed that the dysregulation of NK activity and of cytokine release by NK cells in AD could contribute to neurodegeneration, via disrupted release of cortisol, growth hormone, insulin-like growth factors and melatonin [30]. However, the effect if any of lifelong, reduced NK activity on AD risk is unknown.

## Conclusion

The HLA-B7 &* HLA*-Cw*0702 alleles, which are in tight linkage disequilibrium, are associated with AD in the Oxford population. Homozygotes may be at particular risk. Although surprisingly, this is the largest study to date of the association of *HLA-B *&* C *alleles with AD, a much larger, probably collaborative study will be needed fully to examine the association with homozygosity. If that association is confirmed, further studies will be needed to provide an explanation, including the possible role of low NK cell activity. The association is geographically specific. That may be partly due to differences in linkage disequilibrium with other genes or variable sites. There are many, highly polymorphic loci in the region, including those in retroelements, some of which may interfere with the transcription of nearby genes [32]. The geographical specificity may also be due to different interactions in different populations with environmental, genetic or epigenetic factors.

## Abbreviations

AD, Alzheimer's disease; APOE, apolipoprotein E; CAMCOG, Cambridge Cognitive Examination; CERAD, The Consortium to Establish a Registry for Alzheimer's Disease; CI, confidence interval; HLA, human leukocyte antigen; NINCDS-ADRDA, National Institute of Neurological, Communicative Diseases and Stroke-Alzheimer's Disease and Related Diseases Association; NK, natural killer; OPTIMA, Oxford Project to Investigate Memory and Ageing; PCR, polymerase chain reaction; SSP, sequence-specific primers; TNF, tumour necrosis factor

## Competing interests

The author(s) declare that they have no competing interests.

## Authors' contributions

All authors contributed to the design of the study and approved the final draft. In addition, MB, SF, RH and SB gave expert advice on the *HLA *region and on search strategies in the region; MB supervised the *HLA *genotyping and was responsible for quality control; IQ and AS performed the *HLA *genotyping; DRW isolated the DNA and performed the *APOE *genotyping; LB supplied all the background data on the OPTIMA cohort; DJL was responsible for the data analysis and drafted the manuscript; MB, SB and ADS also made important contributions to the final draft.
